# Scrub typhus with hemorrhagic stroke: a case report

**DOI:** 10.1186/s13256-024-04667-0

**Published:** 2024-07-27

**Authors:** Om Prakash Bhatta, Sabita Chand, Hemant Chand, Prashant Bhetwal, Sachin Awasthi, Aruna Acharya, Ram Chandra Poudel

**Affiliations:** 1 Department of Internal Medicine, Nova Hospital, Dhangadhi, Nepal; 2 Panchkhal PHC, Kavrepalanchowk, Nepal; 3 Department of Emergency Medicine, Yashoda Hospital, Banke, Nepal; 4 Department of Community Medicine, Nepalgunj Medical College, Banke, Nepal

**Keywords:** Scrub typhus, Cerebral hemorrhage, Stroke, Delayed diagnosis, Rare diagnosis

## Abstract

**Background:**

Scrub typhus, caused by *Orientia tsutsugamushi*, rarely leads to central nervous system involvement. Although intracerebral bleeding is rare due to endemicity and a significant proportion of underdiagnoses, it should be considered a noteworthy differential diagnosis in endemic regions in patients with relevant history and clinical findings.

**Case presentation:**

We present the case of a 40-year-old Nepali woman who visited the emergency department with complaints of left-sided weakness for 6 hours and an acute febrile illness with an eschar for 7 days and was diagnosed with scrub typhus by immunoglobulin M enzyme-linked immunosorbent assay of the serum. Imaging revealed a right-sided frontotemporal hematoma, and further examination revealed pulmonary edema with multiple organ dysfunction syndrome. The patient was mechanically ventilated and was treated with antibiotics, steroids, vasopressors, and antipyretics. However, the hematoma was treated conservatively, with ongoing neurological recovery at the 6-month follow-up.

**Conclusion:**

Although neurological complications and intracranial hemorrhage are uncommon, physicians must be cautious when making differential diagnoses and initiating appropriate therapies to avoid serious or fatal complications.

## Background

Scrub typhus, also known as tsutsugamushi disease, is a life-threatening zoonotic disease caused by an obligate intracellular Gram-negative bacillus, *Orientia tsutsugamushi*, that commonly affects farmers in endemic regions in and around the monsoon [1]. It can present with nonspecific illnesses such as fever, headache, myalgia, nausea, vomiting, dizziness, maculopapular rashes, or severe multiorgan dysfunction involving almost any organ system [2, 3].

It is estimated that scrub typhus threatens one billion people globally and leads to at least one million clinical cases annually in the Asia–Pacific region [4]. It is an endemic infection in Nepal with a seroprevalence of 12.2% in patients with acute febrile illness; however, some studies report even higher prevalence rates, as high as 40.3% [5, 6]. However, nationwide data on the genetic diversity of this pathogen in Nepal are lacking.

There is a paucity of literature on the neurological manifestations of scrub typhus. A literature search on intracranial hemorrhage (ICH) associated with scrub typhus showed it to be quite rare. We present the case of a 40-year-old Nepali woman with fever diagnosed with scrub typhus who developed intracranial hemorrhage. This case highlights the need for heightened vigilance among clinicians and greater scrutiny of patients with multisystemic involvement, focusing on tropical infections in endemic regions.

## Case presentation

A 40-year-old Nepali female from the far western region of Nepal presented to our emergency department with sudden onset weakness in the left side of her body for the last six hours. She had difficulty speaking for the same duration. However, the patient had no history of loss of consciousness or comprehension. She developed a fever 7 days prior, which was moderate to high grade, continuous, and without chills or rigor. It was initially associated with mild headaches, multiple episodes of nonbilious vomiting, and generalized body weakness but not with vision difficulty, altered mentation, cough, chest pain, abdominal pain, or burning urine. Her symptoms did not resolve even after 4 days of over-the-counter medication. For the last 3 days, she had shortness of breath and cough with occasional mucoid expectoration.

**Fig. 1:**
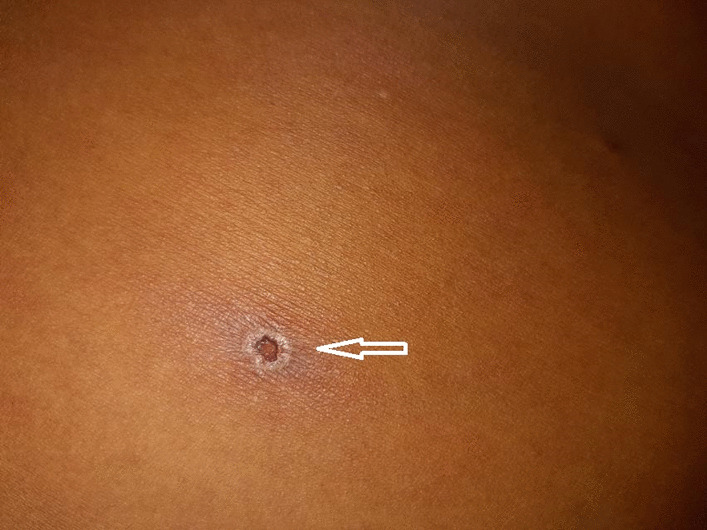
Eschar present in the right buttock region (white arrow)

On initial assessment, the patient was confused and anxious with a Glasgow coma scale (GCS) of E4 V4 M6 (14/15), a temperature of 102 °F, blood pressure (BP) of 80/50 mmHg, heart rate of 123 beats per minute, and oxygen saturation (SPO_2_) of 78% at room air. She was pale and icteric with bilateral chest crepitations. Head-to-toe examination revealed a rash with a brownish-black scab in the right buttock region (Fig. 1). The pupils were reactive to light, bilateral plantar responses were normal, and there was no neck rigidity. The past medical and surgical history was unremarkable.

**Fig. 2:**
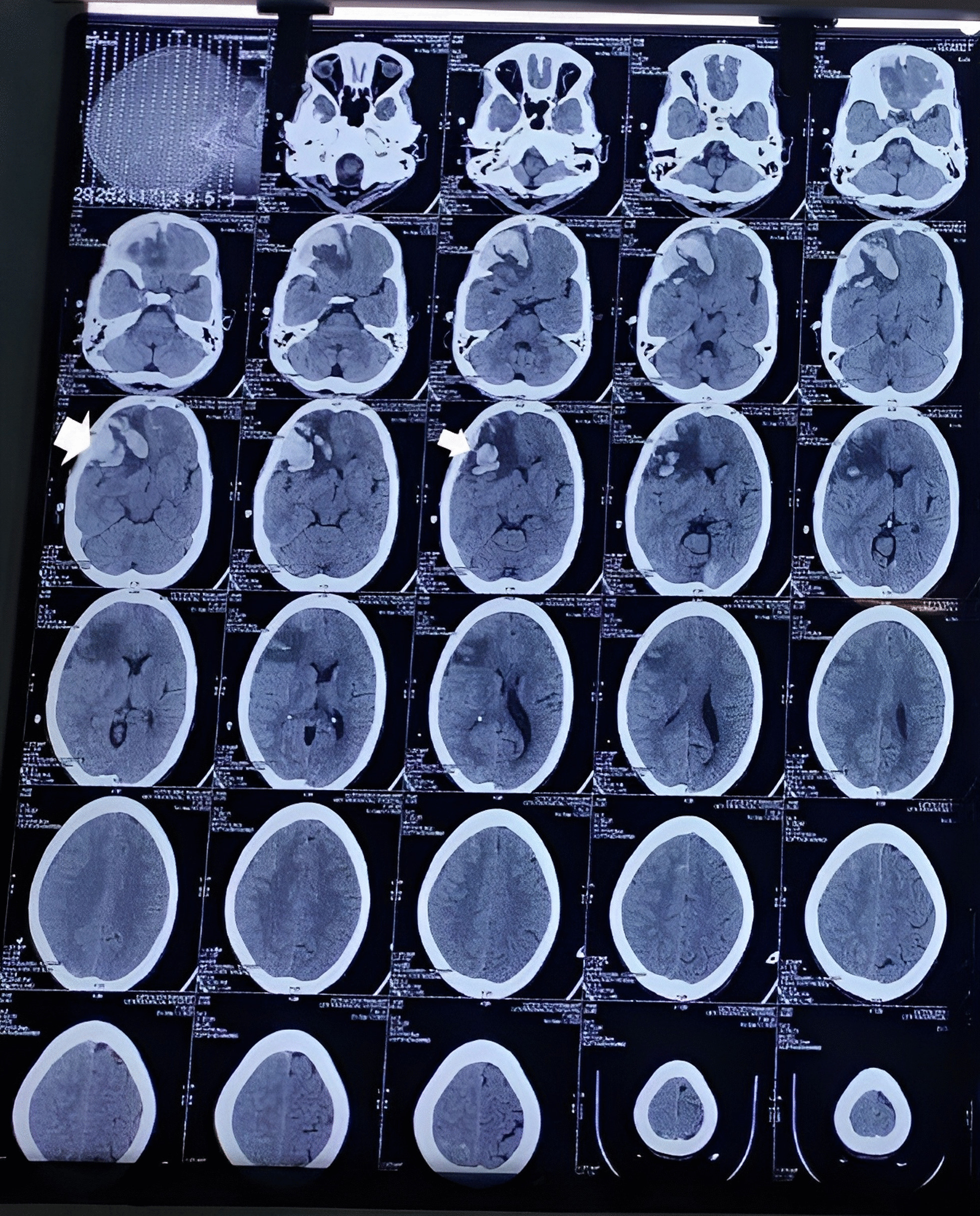
Noncontrast computed tomography head revealed an acute parenchymal hemorrhage in the right frontal lobe (white arrows) with vasogenic edema and mild mass effects

**Fig. 3:**
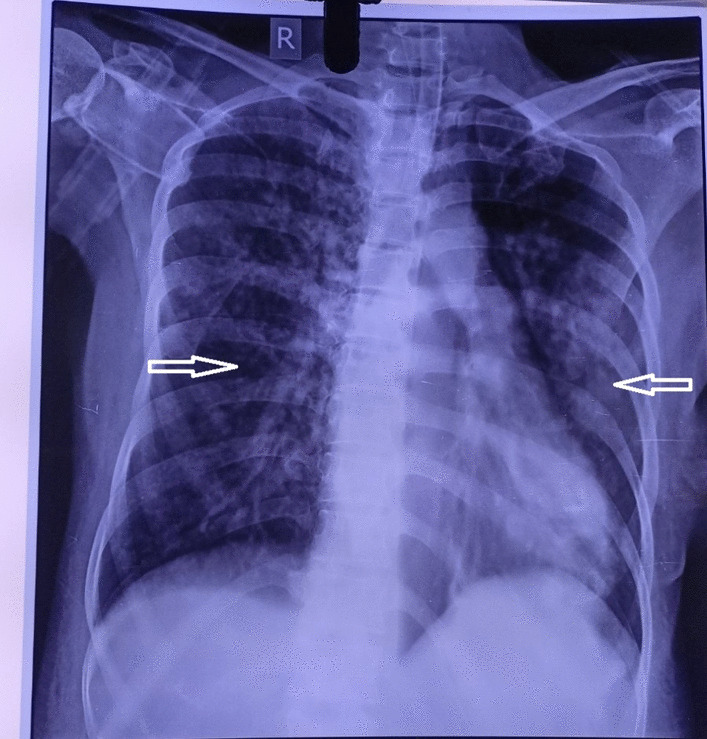
Chest radiograph revealed bilateral pulmonary infiltrates (white arrows)

A noncontrast computed tomography (CT) scan of the head revealed acute parenchymal hemorrhage in the right frontal lobe with vasogenic edema and mild mass effects (Fig. 2). Serological tests were performed for dengue, leptospirosis, and *Brucella*, which were reported to be negative. Tuberculosis was excluded based on negative sputum acid-fast bacilli (AFB) staining and sputum culture results, and human immunodeficiency (HIV) serology was negative. Similarly, thick and thin smears for malaria and polymerase chain reaction (PCR) for coronavirus disease 2019 (COVID-19) were also negative. However, the scrub typhus test was positive for IgM ELISA (Scrub Typhus Detect™ IgM ELISA Kit by InBios). Ultrasonography (USG) of the abdomen and pelvis revealed diffuse gallbladder thickening, borderline splenomegaly, and pleural effusion (right > left). Chest radiography at admission revealed bilateral pulmonary infiltrates and features suggestive of pulmonary edema (Fig. 3).

Laboratory investigations revealed a total leukocyte count (TLC) of 13,200 cells/μL, neutrophils (N) 88%, lymphocytes (L) 5%, monocytes (M) 4%, hemoglobin 10 g/dL, platelets 660,000 cells/μL, random blood sugar 71.2 mg/dL, blood urea 80 mg/dL, serum creatinine 1.7 mg/dL, and serum sodium 134.5 mg/dL. A liver function test showed bilirubin total 6.2 mg/dL and direct 2.7 mg/dL, alanine transaminase 72.3 units/L, aspartate transaminase 228.6 units/L, and alkaline phosphatase 619.9 IU/L. Arterial blood gas (ABG) results were as follows: pH 7.348, paO_2_ 96 mmHg, paCO_2_ 29.5 mmHg, bicarbonate 17.3 mmol/L, and lactate 2.02 mmol/L. The blood cultures were sterile. The detailed laboratory investigations are presented in Table 1.

**Table 1:** Summary of laboratory investigations during hospital stay and their values

Test	Test of the timing	Value	Remarks
Arterial blood gas (ABG)	At admission	pH 7.26, pCO_2_ 31 mmHg, HCO_3_ 15.8 mmol/L, lactate 5.54 mmol/L	
	48 hours after admission	pH 7.53, pCO_2_ 24 mmHg, HCO_3_ 23.3 mmol/L, lactate 1.12 mmol/L	
Liver function test (LFT)	At admission	Bilirubin; total 6.2 mg/dL and direct 2.7 mg/dL alanine transaminase (ALT) 72.3 units/L; aspartate transaminase (AST) 228.6 units/L; alkaline phosphatase (ALP) 619.9 IU/L	
Complete blood count (CBC)	At admission	Hb 10 g/dL, TLC 13,200 cells/μL N88% L5%, platelets 460,000 cells/μL	
	At discharge	Hb 10 gm%, TLC 11,300/cells/μL N86% L8%, platelets 380,000 cells/μL	
Renal function test (RFT)	At admission	Na 134.5 mg/dL, K 3.30 mg/dL, urea 80 mg/dL, creatinine 1.7 mg/dl	
	At discharge	Na 142 mg/dL, K 4.50 mg/dL, urea 46 mg/dL, creatinine 0.8 mg/dL	
PT/INR	At admission	17 seconds/1.38	
Erythrocyte sedimentation rate (ESR)/ c-reactive protein (CRP)	At admission	ESR: 55 mm per hour/CRP: 125 mg/L	

*ABG* arterial blood gas, *CBC* complete blood count, *RFT* renal function test, *PT/INR* prothrombin time/international normalized ratio, *Hb* hemoglobin, *TLC* total leukocyte count, *Na* sodium, *K* potassium, *ESR* erythrocyte sedimentation rate, *CRP* c-reactive protein

Based on brain imaging and serological findings, the working diagnosis was scrub typhus with multiple organ dysfunction syndrome and intracranial hemorrhage. The primary differential diagnoses were dengue fever, falciparum malaria, leptospirosis, and COVID-19.

After the initial evaluation, the patient was transferred to the intensive care unit (ICU) and managed with inotropic support, antipyretics, antibiotics (Piperacillin–tazobactam injection and doxycycline injection), nebulization, and other supportive measures. Initially, oxygen saturation was maintained at 10 L/minute via a nonrebreather face mask (NRBFM). However, her condition deteriorated within an hour, and her oxygen requirement increased, requiring 15 L/minute of oxygen via the NRBFM. Diuretics (torsemide injection) were administered to treat the pulmonary edema, and inotropic support was continued. The patient was mechanically ventilated on the second day of admission because of respiratory distress and a fall in GCS score (during intubation, E3V2M5). She was scheduled for conservative management of the ICH after neurosurgical consultation.

Following 3 days of mechanical ventilation and conservative treatment for ICH, she was extubated on the third day with good recovery of her respiratory function, resolution of multiorgan dysfunction (MODS), and maintenance of saturation of 92–94% with oxygen support of 2–3 L/minute via nasal prongs.

Her neurological status after extubation was good with residual left hemiparesis. The patient began maintaining saturation in room air on the sixth day of admission, and was discharged on the tenth day after being advised to seek physiotherapy. During her recent follow-up after 6 months, she recovered well, with a recent neurological examination showing residual left hemiparesis.

## Discussion

Scrub typhus is transmitted by the bite of the “chigger” larva of the trombiculid mite, which is both a reservoir and vector of the disease [3]. The site of the chigger bite develops localized necrosis of the tissue, producing a black scab called “eschar,” which is pathognomonic of the disease but only identified in approximately one in five cases [7].

Scrub typhus may present with a wide range of clinical features, ranging from acute febrile illness to MODS. Although relatively uncommon, neurological involvement can occur in approximately 20% of patients, with reported manifestations including aseptic meningitis, meningoencephalitis, acute hearing loss, cerebral infarction, polyneuropathy, transverse myelitis, isolated cranial nerve palsy, Landry–Guillain‒Barré syndrome (LGBS), and posterior reversible encephalopathy syndrome (PRES) [3, 4].

Although there is evidence that the blood‒brain barrier may be directly breached by microvascular endothelial damage or by bacteria migrating across cells, either independently or as a result of being engulfed by macrophages, the precise mechanism remains unclear [8]. Entry into the CNS is followed by the activation of transcription factors, such as nuclear factor-kappa B, which induces inflammation and is responsible for various neurological sequelae [9]. During the illness, hemostatic and fibrinolytic changes occur [8]. The bacterium is distributed throughout the body via blood and lymphatics, inducing a vasculitis-type reaction with endothelial injury, perivascular infiltration of leukocytes, increased vascular permeability, and microvascular thrombosis, resulting in end-organ damage [9].

Suspicion of CNS involvement stems from a history of headache, vomiting, altered sensorium, abnormal body movements, dizziness, hearing loss, and urine and stool incontinence. However, systemic manifestations are more frequent in patients with CNS involvement, leading to diagnostic dilemmas [3, 7].

We diagnosed scrub typhus using IgM ELISA (Scrub Typhus Detect™ IgM ELISA Kit), which has a sensitivity of 91.5% and specificity of 92.4% [10]. PCR is widely recommended as a confirmatory test (sensitivity of 90% and specificity of 100%); however, it was unavailable in our setting [11]. The presence of bacteria in the cerebrospinal fluid (CSF) can be demonstrated using nested PCR [12].

Routine blood tests and other specialized investigations can also be performed depending on systemic involvement. Although there are no specific neuroradiological features pathognomonic of scrub typhus, supportive imaging investigations include computed tomography (CT), magnetic resonance imaging (MRI), and CT angiography to diagnose and confirm intracranial hemorrhage [7, 13].

This patient with an acute febrile illness had features suggestive of stroke. Other common causes of stroke were excluded from the history, as she was not on any anticoagulants or other medications and had no other comorbidities or family history of stroke. Additionally, other infectious causes of stroke, such as HIV, HSV, tuberculosis, dengue fever, falciparum malaria, and leptospirosis, were excluded [7].

The median mortality rate of untreated scrub typhus is approximately 6%, depending on organ involvement and age of the patient [14]. Doxycycline is the antibiotic of choice and shows an excellent response within 48 hours of administration, when aided by supportive measures. In pregnancy, doxycycline is contraindicated, and azithromycin is used [7, 15].

Concurrently, patients require management of hemorrhagic stroke and neurosurgical evaluation and management to assess mass effects. Blood pressure control is crucial, and antiepileptic drugs may be used for seizure prophylaxis. Rehabilitation and long-term care may be needed to address deficits and aid recovery once a patient’s condition stabilizes [3, 16].

Chung *et al.* reported three patients diagnosed with scrub typhus through serology and PCR who experienced delayed administration of effective antibiotics after the appearance of symptoms and presented with a cerebrovascular accident in the late acute phase, resulting in fatality [12].

## Conclusion

Clinicians should be aware of the diverse manifestations and severe complications of scrub typhus, particularly in and around the monsoon in endemic regions. Although rare, it may present with life-threatening neurological manifestations that can mimic other infectious pathologies. A precise history, thorough clinical examination, and necessary investigations help reach a final diagnosis and provide optimal management. Timely management with antimicrobial agents leads to a good response with little residual neurological dysfunction.

## Data Availability

The authors declare that data supporting the findings of this study are available within the article.

## Abbreviations

CNS: Central nervous system
CT: Computed tomography
MRI: Magnetic resonance imaging
MODS: Multiorgan dysfunction
ABG: Arterial blood gas
GCS: Glasgow coma scale
LGBS: Landry–Guillain‒Barré syndrome
PRES: Posterior reversible encephalopathy syndrome
IgM: Immunoglobulin M
ELISA: Enzyme-linked immunosorbent assay

## References

[1] Misra UK, Kalita J, Mani VE. Neurological manifestations of scrub typhus. J Neurol Neurosurg Psychiatry. 2015;86(7):761–6. 25209416 10.1136/jnnp-2014-308722

[2] Rana A, Mahajan SK, Sharma A, Sharma S, Verma BS, Sharma A. Neurological manifestations of scrub typhus in adults. Trop Doct. 2017;47(1):22–5. 27059055 10.1177/0049475516636543

[3] Basu S, Chakravarty A. Neurological manifestations of scrub typhus. Curr Neurol Neurosci Rep. 2022;22(8):491–8. 35727462 10.1007/s11910-022-01215-5

[4] Matos AL, Curto P, Simões I. Moonlighting in Rickettsiales: expanding virulence landscape. Trop Med Infect Dis. 2022;7(2):32. 35202227 10.3390/tropicalmed7020032 PMC8877226

[5] Pokhrel A, Rayamajhee B, Khadka S, Thapa S, Kapali S, Pun SB, *et al*. Seroprevalence and clinical features of scrub typhus among febrile patients attending a referral hospital in Kathmandu. Nepal Trop Med Infect Dis. 2021;6(2):78. 34068402 10.3390/tropicalmed6020078 PMC8163188

[6] Gautam R, Parajuli K, Tshokey T, Stenos J, Sherchand JB. Diagnostic evaluation of IgM ELISA and IgM Immunofluorescence assay for the diagnosis of Acute Scrub Typhus in central Nepal. BMC Infect Dis. 2020;13(20):138. 10.1186/s12879-020-4861-y PMC7020552 32054525

[7] Alam AM, Gillespie CS, Goodall J, Damodar T, Turtle L, Vasanthapuram R, *et al*. Neurological manifestations of scrub typhus infection: a systematic review and meta-analysis of clinical features and case fatality. PLoS Negl Trop Dis. 2022;16(11): e0010952. 36441812 10.1371/journal.pntd.0010952 PMC9731453

[8] Kim HC, Yoon KW, Yoo DS, Cho CS. Hemorrhagic transformation of scrub typhus encephalitis: a rare entity. Clin Neuroradiol. 2015;25(4):415–8. 25373351 10.1007/s00062-014-0348-9

[9] Gaba S, Garg S, Gupta M, Gupta R. Haemorrhagic encephalitis in the garb of scrub typhus. BMJ Case Rep. 2020;13(8): e235790. 32859623 10.1136/bcr-2020-235790 PMC7454241

[10] Blacksell SD, Kingston HWF, Tanganuchitcharnchai A, Phanichkrivalkosil M, Hossain M, Hossain A, *et al*. Diagnostic accuracy of the InBios Scrub Typhus Detect™ ELISA for the detection of IgM Antibodies in Chittagong, Bangladesh. Trop Med Infect Dis. 2018;3(3):95. 30274491 10.3390/tropicalmed3030095 PMC6160969

[11] Kim CM, Cho MK, Kim DM, Yun NR, Kim SW, Jang SJ, *et al*. Accuracy of conventional PCR targeting the 16S rRNA gene with the Ot-16sRF1 and Ot-16sRR1 primers for diagnosis of scrub typhus: a case-control study. J Clin Microbiol. 2016;54(1):178–9. 26491185 10.1128/JCM.01754-15 PMC4702754

[12] Chung JH, Yun NR, Kim DM, Lee JW, Yoon SH, Kim SW. Scrub typhus and cerebrovascular injury: a phenomenon of delayed treatment? Am J Trop Med Hyg. 2013;89(1):119–22. 23716407 10.4269/ajtmh.13-0094 PMC3748467

[13] Jeong YJ, Kim S, Wook YD, Lee JW, Kim KI, Lee SH. Scrub typhus: clinical, pathologic, and imaging findings. Radiographics. 2007;27(1):161–72. 17235005 10.1148/rg.271065074

[14] Taylor AJ, Paris DH, Newton PN. A systematic review of mortality from untreated scrub typhus (Orientia tsutsugamushi). PLoS Negl Trop Dis. 2015;9(8): e0003971. 26274584 10.1371/journal.pntd.0003971 PMC4537241

[15] Kim YS, Lee HJ, Chang M, Son SK, Rhee YE, Shim SK. Scrub typhus during pregnancy and its treatment: a case series and review of the literature. Am J Trop Med Hyg. 2006;75(5):955–9. 17123995 10.4269/ajtmh.2006.75.955

[16] Srinivasa Murthy CL, Namitha P, Raghavendra K, Kumar N, Pejaver R. An unusual case of typhus group rickettsial infection presenting as cerebrovascular stroke. Pediatr Infect Dis. 2015;7(3):74–7.

